# Application of EvaGreen for the assessment of *Listeria monocytogenes* АТСС 13932 cell viability using flow cytometry

**DOI:** 10.3934/microbiol.2019.1.39

**Published:** 2019-01-25

**Authors:** Elena Kotenkova, Dagmara Bataeva, Mikhail Minaev, Elena Zaiko

**Affiliations:** V.M. Gorbatov Federal Research Center for Food Systems of RAS, 109316, Talalikhina St., 26, Moscow, Russia

**Keywords:** EvaGreen, flow cytometry, viability, *Listeria monocytogenes*

## Abstract

Determination of eukaryotic cell viability using flow cytometry is widespread and based on the use of fluorescent dyes such as SYTO, DAPI, SYBR, PI, and SYTOX. For many years, traditional microbiological methods have been used to successfully analyze prokaryotic cells, but the application of flow cytometry should be considered because it provides an opportunity for quantitative assessment. A combination of SYTO 9 or SYBR green and PI has been used successfully. DNA-binding dyes such as SYTO 9, SYBR green, and EvaGreen are used in qPCR. The aim of this study was to assess the feasibility of EvaGreen to determine the viability of *Listeria monocytogenes* АТСС 13932 cells using flow cytometry. RNA from *Escherichia coli* ATCC 25922 was isolated using the MagNA Pure LC RNA Isolation Kit-High Performance (Roche, Germany) according to the kit instructions on MagNA Pure LC® 2.0 (Roche, Switzerland). Chicken DNA was isolated using the Sorb-GMO-B kit (Syntol CJSC, Russia) according to the kit instructions. RNA from *E. coli* ATCC 25922, chicken DNA, a positive control, and a negative control of *L. monocytogenes* АТСС 13932 were stained with EvaGreen and analyzed on the Guava EasyCyte flow cytometer (Merck Millipore, Germany). Chicken DNA demonstrated both green and red fluorescence, while *E. coli* RNA displayed only red fluorescence. While the positive *L. monocytogenes* АТСС 13932 control and chicken DNA demonstrated similar fluorescence properties, the negative control showed a localization similar to that observed with *E. coli* RNA. Degraded ssDNA and RNA stained with EvaGreen demonstrated red fluorescence. Although EvaGreen is a class III dye, we observed fluorescence of live *L. monocytogenes* АТСС 13932 cells in the positive control stained with EvaGreen. The observed phenomenon was linked to the solution composition. It is necessary to repeat this analysis with various solution compositions as well as a wide range of both Gram-positive and Gram-negative bacteria to determine the effects on cell envelope permeability of EvaGreen.

## 1. Introduction

Determination of cell viability is essential for *in vitro* studies. Approaches for the evaluation of live/dead cells such as flow cytometry and microscopy use fluorescent dyes, but flow cytometry generates numeric values immediately. A wide range of dyes are available, and their ability to stain depends on vital properties such as cell membrane integrity (e.g. PI, SYTOX, TOTO), cell membrane potential (e.g. Rh123, DiBAC4), membrane pump activity (e.g. CFDA, Rh123), and enzymatic activity (e.g. CFDA, Calcein-AM, ChemChrome). In addition, permanent DNA stains (e.g. SYTO, DAPI, SYBR) are also available [1]. Eukaryotic cells generally require the use of only one dye when assays are performed on a flow cytometer. Using side and forward scatter signals, an operator can determine the population of whole homogeneous cells with similar size and granularity. DNA-binding dyes such as SYTO and DAPI are commonly used to differentiate between live and dead cells, and PI is commonly used for the evaluation of cell membrane integrity. These dyes are usually applied separately, and other parameters are calculated by the operator or automatically (e.g. with commercial kits) because the count of the general cell population is already known.

Determination of prokaryotic cell viability by flow cytometry is more difficult. If the cytometer detector is poor, it is impossible to count cells using side and forward scatter signals. Therefore, it is necessary to apply a combination of dyes with emissions of different wavelengths to count live and dead cells separately. Combining DNA-binding dyes with those used to evaluate cell membrane integrity is the most common method (e.g. the combination of SYTO 9 and PI). While SYTO 9, with a green emission, enters all cells and binds to their DNA, PI, which fluoresces red after binding to DNA, only enters cells with damaged membranes [2],[3]. Nevertheless, Stiefel et al., 2012 noted that in addition to background fluorescence and the cross-signaling of these dyes, SYTO 9 causes bleaching effects and has different binding affinities to live and dead cells [4]. Therefore, there is a need for studies on alternative dyes. Moreover, the quantitative assessment of prokaryotic viability is essential, especially for the confirmation of activity of novel antimicrobial substances. Traditional microbiological methods have long been used successfully for these purposes. However, disk and agar well diffusion methods, agar and broth dilution tests for minimum inhibitory concentration (MIC) determination, Etests and Time-kill tests [5]–[8], bioautography [9],[10], and qPCR [11] mainly provide descriptive information. Therefore, the development of an effective, alternative approach using flow cytometry for the evaluation of prokaryotic cell viability is a necessity. Some dyes used in PCR analysis have also been used for the assessment of bacterial viability. It has also been shown that a combination of SYBR green and PI was more successful than with SYTO 9 [12]. Therefore, the aim of this study was to assess the feasibility of EvaGreen in determining the cell viability of *Listeria monocytogenes* АТСС 13932 and *Staphylococcus aureus* АТСС 25923 using flow cytometry.

## 2. Materials and methods

### 2.1. Sample preparation

#### 2.1.1. Isolation of chicken DNA

Meat samples were ground with a Retsch GM200 blade homogenizer (Retsch, Germany). A total of 50 ± 1.0 mg was collected for DNA isolation. DNA was isolated using a Sorb-GMO-B kit (Syntol CJSC, Russia) according to the instructions. Samples were mixed with 800 µL of lysis buffer and 15 µL of proteinase K, incubated for 60 min at 60 °C, and centrifuged on MiniSpin columns (Eppеndorf, Germany) at 13000 rpm for 5 min. Supernatants were transferred to new tubes, and 500 µL of extraction solution was added. Then, mixtures were intensively mixed and centrifuged on MiniSpin columns (Eppеndorf, Germany) at 13000 rpm for 10 min. A total of 300 µL of supernatant was transferred to new tubes and mixed with 625 µL of precipitating solution and sorbent (24:1). Then, mixtures were intensively mixed for 10 min and centrifuged on MiniSpin columns (Eppеndorf, Germany) at 7000 rpm for 1 min. Precipitates were washed three times by the addition of 500 µL of wash solution, intensively mixed for 2 min, and centrifuged on MiniSpin columns (Eppеndorf, Germany) at 7000 rpm for 0.5 min. Precipitates were dried at 60 °C for 5 min; then, 200 µL of buffer was added, samples were mixed, incubated at 60 °C for 10 min, and centrifuged on MiniSpin columns (Eppеndorf, Germany) at 13000 rpm for 2 min. A total of 150 µL of supernatant was taken for further testing.

#### 2.1.2. Isolation of *Escherichia coli* ATCC 25922 RNA

The *Escherichia coli* ATCC 25922 strain was obtained from the State Research Center for Applied Biotechnology and Microbiology (Obolensk, Moscow region, Russia). RNA from *E. coli* ATCC 25922 was isolated using a MagNA Pure LC RNA Isolation Kit-High Performance (Roche, Germany) according to the instructions. Cells were lysed by incubation with a special buffer containing chaotropic salt, and proteinase K digestion destroyed the remaining proteins and nucleases. Then, magnetic glass particles (MGPs) were added to bind RNA, and DNA was degraded with DNase. Unbound substances were removed by several wash steps, and the purified RNA was eluted using elution buffer. Cells were centrifuged for 10 minutes at 1500 rpm on MiniSpin columns (Eppеndorf, Germany), resuspended in 200 µL of phosphate buffered saline (PBS), and transferred into a well of the sample cartridge. Sample cartridges were placed on the reagent/sample stage, and the “RNA HP Cells” protocol was started. The isolation was performed on a MagNA Pure LC® 2.0 (Roche, Switzerland).

#### 2.1.3. Positive and negative *Listeria monocytogenes* АТСС 13932 controls

*L. monocytogenes* АТСС 13932 and *Staphylococcus aureus* АТСС 25923 strains were obtained from the State Research Center for Applied Biotechnology and Microbiology (Obolensk, Moscow region, Russia). Cultures were grown on slanted Trypticase soy agar (TSA, Liofilchem) at 37 °C for 24 h. Cultures from the agar surface were removed with normal saline solution and adjusted to a concentration of 1 × 10^6^ cells/mL according to the standard of McFarland turbidity. A total of 100 µL of the suspensions was transferred to Eppendorf tubes and mixed with 2 mL of Trypticase soy broth (TSB, Liofilchem) and incubated on a thermoshaker TS-100 (BioSan, Latvia) at 30 °C for 4 h. Suspensions were centrifuged on MiniSpin columns (Eppеndorf, Germany) at 5000 rpm for 5 min. Cell precipitates were washed three times with normal saline solution followed by centrifugation. Cell suspensions at an approximate concentration of 1 × 10^6^ cells/mL were prepared in normal saline solution according to the McFarland turbidity standard. Suspensions with an approximate concentration of 1 × 10^6^ cells/mL were used as positive controls. To obtain negative control-1 of *L. monocytogenes* АТСС 13932 and *S. aureus* АТСС 25923, the resulting suspensions were heated at 100 °C for 10 min. To obtain negative control-2 of *L. monocytogenes* АТСС 13932, cell pellets were washed with 70% isopropyl alcohol and resuspended at an approximate concentration of 1 × 10^6^ cells/mL.

### 2.2. Flow cytometry analysis procedure

#### 2.2.1. DNA and RNA analysis protocol

A total of 5 µL of DNA/RNA was mixed with 5 µL EvaGreen (Synthol, Russia), 380 µL of deionized water, and 10 µL of DMSO (Biolot, Russia); then, samples were incubated in the dark for 15 min and green and red fluorescence signals were measured on a Guava EasyCyte flow cytometer (Merck Millipore, Germany) up to 5000 events.

#### 2.2.2. *L. monocytogenes* АТСС 13932 analysis protocols

(1) Protocol 1: A total of 20 µL of *L. monocytogenes* АТСС 13932 cells or *S. aureus* АТСС 25923 cells was mixed with 5 µL of EvaGreen (Synthol, Russia), 365 µL of deionized water, and 10 µL of DMSO (Biolot, Russia); then, samples were incubated in the dark for 15 min and green and red fluorescence signals were measured on a Guava EasyCyte flow cytometer (Merck Millipore, Germany) up to 5000 events. To prepare mixed samples, a 10-µL positive control (live cells) and a 10-µL negative control-1 (dead cells killed by heating) were used.

(2) Protocol 2: A total of 20 µL of *L. monocytogenes* АТСС 13932 cells was mixed with 5 µL of EvaGreen (Synthol, Russia) and 375 µL of 0.9% NaCl solution (Panreac, Spain); then, the samples were incubated in the dark for 15 min and green and red fluorescence signals were measured on a Guava EasyCyte flow cytometer (Merck Millipore, Germany) up to 5000 events.

## 3. Results

Parameters of analysis are presented in Table 1. Low gain on the green channel was used for chicken DNA analysis due to the primary intense fluorescence when bound to EvaGreen. Low gain on the red channel was used for *E. coli* RNA measurement. Compensation coefficients were applied to obtain better separation between live and dead cells in *L. monocytogenes* samples.

**Table 1.** Parameters of analysis.

Sample ID	Gains					Compensation
	FSC	SSC	GRN	YEL	RED	GRN-%RED
Chicken DNA	5.2	1.9	8.0	12.3	6.7	0.0
*Escherichia coli* RNA	1.7	3.1	20.7	12.3	3.5	0.0
*Listeria monocytogenes* АТСС 13932 (positive control)	5.2	1.9	23.6	12.3	8.7	3.6
*Listeria monocytogenes* АТСС 13932 (negative control-1)	5.2	1.9	23.6	12.3	8.7	5.1
*Listeria monocytogenes* АТСС 13932 (negative control-2)	5.2	1.9	15.3	12.3	8.7	2.2

**Figure 1.**
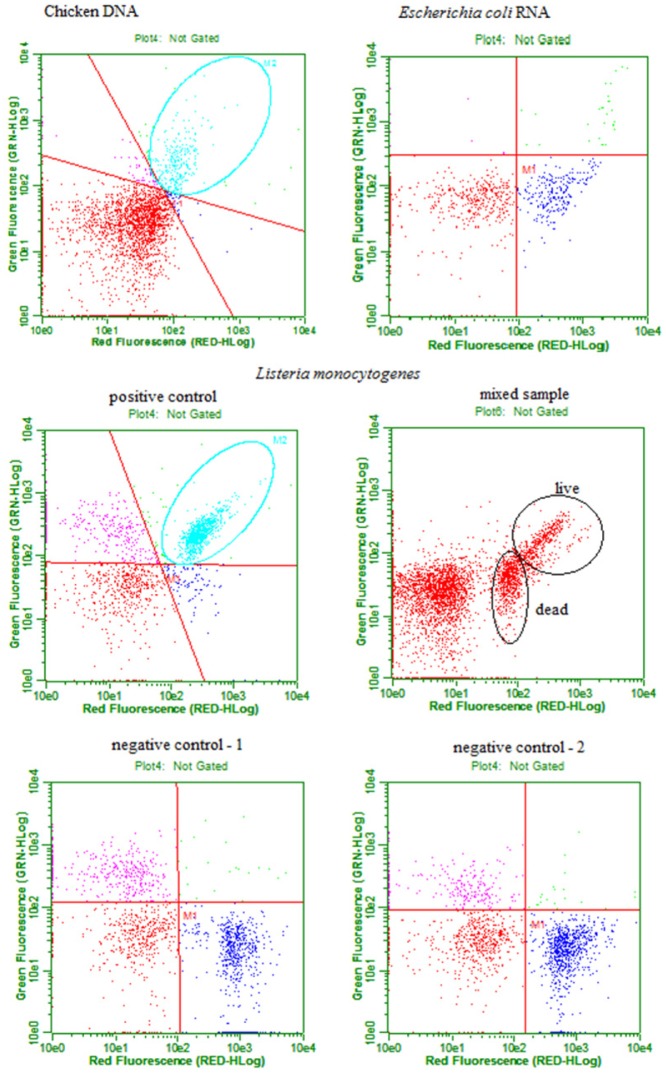
Flow cytometry analysis of DNA, RNA, and *L. monocytogenes* АТСС 13932 according to protocol 1.

Results of the flow cytometry analysis of DNA, RNA, and *L. monocytogenes* АТСС 13932 according to protocol 1 are presented in Figure 1. Both green and red fluorescence were observed in chicken DNA stained with EvaGreen; corresponding counted events are located in the upper right square of the plot. RNA isolated from *E. coli* and stained with EvaGreen displayed only red fluorescence; all counted events are located in the lower right square of the plot. The positive control of *L. monocytogenes* АТСС 13932 displayed a localization similar to chicken DNA; a large number of live cells are located in the upper right square of the plot. Negative controls (1 and 2) of *L. monocytogenes* АТСС 13932 showed a similar localization to *E. coli* RNA; a large number of dead cells are located in the lower right square of the plot. The positive control of *L. monocytogenes* АТСС 13932 displayed a concentration of 2.26 × 10^6^ live cells/mL, negative control 1 displayed a concentration of 2.35 × 10^6^ dead cells/mL, and negative control 2 displayed a concentration of 2.77 × 10^6^ dead cells/mL. To examine the discriminatory ability of EvaGreen, a mixed sample of *L. monocytogenes* АТСС 13932 was prepared according to protocol 1 using 10 µL of positive control and 10 µL of negative control. Both green and red fluorescence were observed in live cells, and corresponding counted events are located in the upper right square of the plot. Only red fluorescence was observed in dead cells, and all counted events are located in the lower right square of the plot. However, the separation was not distinct enough.

## 4. Discussion

**Figure 2.**
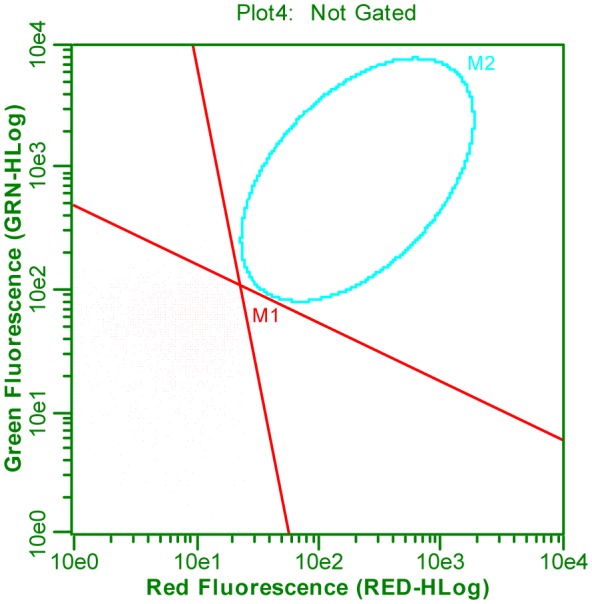
Flow cytometry analysis of *Listeria monocytogenes* АТСС 13932 according to protocol 2.

According to the safety report for EvaGreen® dye [13] and Chiaraviglio & Kirby, 2014 [14], EvaGreen is a non-permeable, nontoxic (class III) dye and appears to be membrane-impermeable; this is evident from the absence of cell nuclear staining with HeLa cells over a 30 min incubation period. Nevertheless, we observed fluorescence of live *L. monocytogenes* АТСС 13932 cells in the positive control stained with EvaGreen. Presumably, the observed phenomenon can be linked to the solution composition; a mixture of DMSO and deionized water was used in the protocol. When a 0.9% NaCl solution was used, no fluorescence was observed in the positive control of *L. monocytogenes* АТСС 13932 stained with EvaGreen (Figure 2).

However, eukaryotic mammalian cells were used for the study of EvaGreen permeability in both reports. Prokaryotic cells have different cell wall structures depending on whether they are Gram-positive or Gram-negative [15]. The type of cell envelope could influence EvaGreen permeability; however, in our study, we only considered the staining of Gram-positive bacteria.

**Figure 3.**
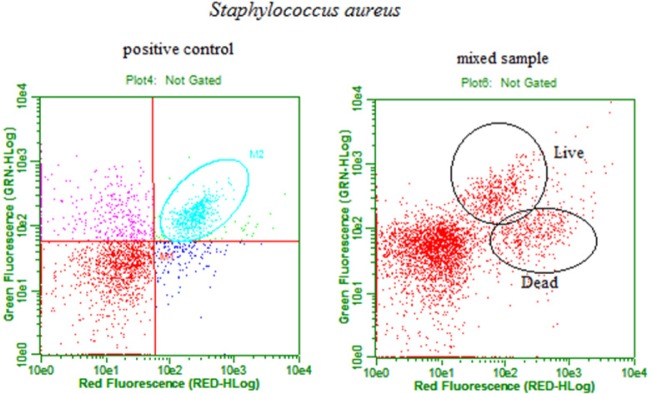
Results of the flow cytometry analysis of *Staphylococcus aureus* АТСС 25923 according to protocol 1.

We also examined *S. aureus* АТСС 25923 staining by EvaGreen (Figure 3). The positive control of *S. aureus* АТСС 25923 displayed a localization similar to *L. monocytogenes* АТСС 13932; a large number of live cells are located in the upper right square of the plot. Mixed samples of *S. aureus* were prepared according to protocol 1 using 10 µL of the positive control and 10 µL of the negative control. Live cells displayed both green and red fluorescence, and corresponding counted events are located in the upper right square of the plot. Dead cells displayed only red fluorescence, and all counted events are located in the lower right square of the plot. The separation was more distinct than in the case of *L. monocytogenes* АТСС 13932.

We also noticed red fluorescence of dead *L. monocytogenes* АТСС 13932 cells in the negative control stained with EvaGreen that was similar to *E. coli* RNA bound to EvaGreen. Presumably, the heat treatment of *L. monocytogenes* АТСС 13932 destroyed cell integrity and led to the conversion of double-stranded DNA to single-stranded DNA [16]. RNA and ssDNA were located in the buffer solution where ssDNA could be degraded by nucleases [17]; therefore, both degraded ssDNA and RNA stained with EvaGreen could demonstrate red fluorescence.

## 5. Conclusions

It was observed that EvaGreen dye, which is commonly used in PCR analysis, stained live cells of *L. monocytogenes* АТСС 13932 and fluoresced in green and red spectra; the dye also stained dead cells and only demonstrated red fluorescence. However, the solvent composition and cell type influenced EvaGreen permeability. Therefore, it is necessary to repeat this analysis on a wide range of both Gram-positive and Gram-negative bacteria. In conclusion, EvaGreen dye has prospective applications in flow cytometry.
